# Laryngismus Stridulus

**Published:** 1852-12

**Authors:** J. W. Bright

**Affiliations:** Louisville, Ky.


### WESTERN JOURNAL

OF

### MEDICINE AND SURGERY.

---

DECEMBER, 1852.

---

*Art.* I. *Laryngismus Stridulus.* By J. W. Bright, M. D., of Louisville, Ky.

This is a disease of the Thymus gland of infants, and takes its name from the stridulous, or crowing, croaking sound in breathing. It assimulates croup and bronchitis in many of its symptoms. And the true character of the disease is detected in its early stage only by applying the ear to the chest of the patient. Delicate or scrofulous children, from the age of three or four months, to three or four years, are most liable to this disease. Males are more subject to it than females. It makes its onset by slight paroxysms of fever, not preceded by any perceptible chill or cold stage. The child appears to have taken cold. The cheeks are flushed, and the pulse is more or less accelerated. The breathing is more hurried than usual, and difficult, owing to the pressure of the enlarged thymus gland upon the trachea. It is attended by a short troublesome cough. The phlegm is loose, but raised with difficulty, and when discharged, is white and frothy. The child is disposed to throw its head back, with its mouth open, and roll its tongue from side to side, with a chewing motion of the jaws. If it is old enough to raise its hand, it will make frequent efforts to thrust its fingers into its mouth. The phlegm appears to be loose and rattles, but no discharge is made by the effort of coughing. The bowels are inclined to be constipated; but when evacuations are obtained, they are not unnatural, except phlegm has been swallowed. The skin is sometimes dry and hot, but generally it is relaxed, and a cool sweat stands on the forehead; after a day or two has elapsed, the superior portion of the sternum is raised, and there is a fulness above the clavicles. The child sleeps but little, owing to the difficulty of breathing. The countenance is anxious, the complexion varies from a glossy paleness to a livid pallor. There is great thirst, and the child will take with avidity anything that is presented to its mouth. The pathology of this disease is often mistaken, and it is treated as for croup or bronchitis: and the patient falls a victim to a misdirected treatment.

*Treatment.*—This, as all other diseases, should be treated as the pathology requires.

*First*, relieve the stomach and bowels by a mercurial cathartic.

*Secondly*, keep down fever by the Spirits of Mindereri, and Spirits of Nitre, in the proportion of seven of the former to three of the latter. This should be given in repeated doses every hour, of ten drops, more or less, according to the age and strength of the patient. At the same time an ointment of the Iodide of Potassa (officinal) should be frequently applied over the sternum and above the clavicles. When the patient is free from fever, a solution of the Hydriodate of Potass, ten grains to the ounce of distilled water, should be given in ten drop doses (more or less, according to the age of the patient,) every two hours. The bowels should be kept open with small and frequently repeated doses of calomel, Ipecac, and Nitre. If the phlegm accumulates, and is not freely discharged, an emetic of alum and honey should be given. This is prepared by mixing one drachm of pulverised alum in one ounce of strained honey. Give a teaspoonful every ten minutes till the child pukes freely, and discharges the phlegm. This emetic may be repeated as often as the circumstances require, as it does not debilitate the child, nor leave nausea after vomiting. If these remedies be persevered in for a few days, the disease will yield and the patient recover. If the skin be hot and dry at any time, the tepid bath may be used with advantage. The child should lie with its shoulders and head a little elevated.—In this position it breathes easier. Before the child is weaned, the breast milk is the only diet necessary for it. After weaning, the diet should be light and thin. No recovery can be effected until the thymus gland is reduced. Great care should be taken after recovery that the child be not exposed to cold or dampness, otherwise it will relapse. At the same time, pure fresh air without exposure, will confirm the cure.

LOUISVILLE, KY., December, 1852.

